# The APC/C and CK1 in the developing brain

**DOI:** 10.18632/oncotarget.4797

**Published:** 2015-07-09

**Authors:** Clara Penas, Mary E. Hatten, Nagi G. Ayad

**Affiliations:** Center for Therapeutic Innovation, Department of Psychiatry and Behavioral Sciences, University of Miami Miller School of Medicine, Miami, FL, USA

**Keywords:** the anaphase promoting complex, neurite out-growth, granule cell progenitors, casein kinase, cell cycle exit

Casein Kinase 1δ (CK1δ) is a serione/threonine kinase required for cell cycle progression, circadian rhythm, vesicle trafficking, and neurite outgrowth [1]. CK1δ is also a therapeutic target in various cancers, Alzheimer's disease, alcoholism, and sleep disorders [1]. To examine the role of CK1δ in brain development, we used cerebellar granule cell progenitors (GCPs) as a model system. GCPs are the most abundant neurons in the mammalian brain and are one of two principal neurons in the cerebellar circuitry [2]. CK1δ is expressed in GCPs during peak times of proliferation (postnatal day 6-postnatal day 8). To probe a role for CK1δ in proliferation of GCPs during this time, we assayed proliferation in GCPs lacking CK1δ, after knockdown of CK1δ by RNAi methodology or in purified GCPs treated with highly specific CK1δ inhibitor [3]. In all three cases,3H-thymidine incorporation assays showed reduced levels of proliferation. Given CK1δ's role in GCP neurogenesis, we anticipated that CK1δ levels would decrease as GCPs exit the cell cycle. Indeed, we found that CK1δ protein but not mRNA levels dropped during cell cycle exit, which suggested that CK1δ is targeted for degradation during this time.

Importantly, biochemical assays demonstrate that CK1δ is targeted for degradation via the Anaphase Promoting Complex/cyclosome (APC/C), a multi-subunit E3 ubiquitin ligase, which has well-established roles in mitotic exit and G1 progression [2]. APC/C is also active in differentiating and differentiated cells [4]. APC/C associates with one of two activators termed Cdc20 or Cdh1, which recruit substrates to bring them into close proximity of the E2 enzyme bound to APC/C [4]. We report that Cdh1 binds to CK1δ to initiate APC/C dependent ubiqutination. *In vitro* ubiquitination assays containing purified APC/C and CK1δ demonstrate that APC/C mediates CK1δ polyubiquitination *in vitro*. APC/C mediate ubiquitination of CK1δ was dependent on two N-terminal destruction boxes in CK1δ as mutation of these sites abrogated ubiquitination *in vitro* [2]. To demonstrate a requirement for CK1δ *in vivo* we deleted CK1δ in GCPs in the cerebellum [2]. Deletion of the APC/C activator Cdh1 in GCPs increased CK1δ levels, suggesting that CK1δ is turned over in GCPs [2]. Collectively, these studies suggest that APC/C targets CK1δ for destruction *in vitro* and *in vivo* and that APC/C^Cdh1^ is an important regulator of GCP proliferation by controlling CK1δ.

Our studies therefore suggest that APC/C-mediated degradation of CK1δ functions in multiple steps in CNS neuronal differentiation. CK1δ has been linked to neurite outgrowth [5] and thus it will be important to determine whether APC/C mediated degradation of CK1δ occurs in axons or dendrites. Prior studies demonstrated that APC/C inhibition in postmitotic neurons [4] increases neurite outgrowth while CK1δ inhibition reduces neurite outgrowth in cell lines [5]. Thus, CK1δ could be one of the substrates, which APC/C targets during neurite outgrowth, and whose levels rise during APC/C inhibition or depletion. It will be important to determine whether CK1δ protein levels are modulated by APC/C active in postmitotic neurons. Interestingly, there are two forms of the APC/C that are active in postmitotic neurons, APC/^CCdh1^ and APC/C^Cdc20^ [4]. APC/C^Cdh1^ represses axonal growth [4] while APC/CCdc20 activity controls dendritic morphogenesis [4]. APC/C^Cdc20^ is localized to centrosomes in postmitotic neurons. Given the finding that CK1δ is localized to centrosomes [5] it will be interesting to determine whether APC/C^Cdc20^ is able to induce CK1δ destruction at centrosomes. An alternative could be that centrosome bound CK1δ is protected from APC/C mediated degradation as other APC/C substrates cannot be ubiquitinated and degraded when bound to microtubules [6].

As centrosomal proteins often have roles in migration it will be important to determine whether APC/C mediated control of CK1δ is linked to migration of neuronal precursors. Consistent with a role for CK1δ in neuronal migration we found that CK1δ inhibition reduced GCP migration *ex vivo* (unpublished observations).

**Figure 1:**
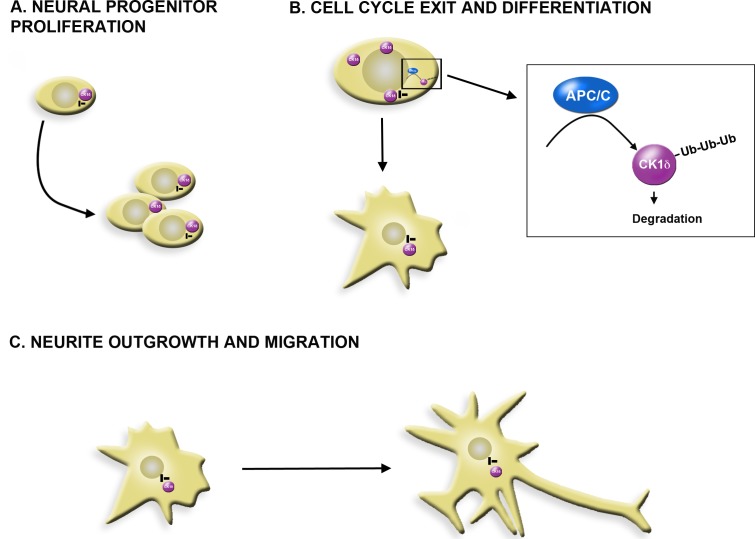
Model of CK1δ and APC/C during proliferation and differentiation of GCPs **A.** CK1δ activity is required for GCP proliferation. **B.** CK1δ is degraded via APC/C mediated ubiquitination during GCP cell cycle exit and differentiation. Some centrosome bound CK1δ may be protected from APC/C mediated degradation. **C.** Centrosomal CK1δ is required for neurite outgrowth and migration.

In addition, since CK1δ has a role in ciliogenesis^1^ and APC/C^Cdc20^ has been reported to be required for primary cilia formation [7], the APC/C may interact with CK1δ in primary cilia. It will be interesting to determine whether APC/C^Cdc20^ induces CK1δ degradation within cilia. Interestingly, since the primary cilium is required for Hedgehog (Hh) pathway signaling as we found that CK1δ inhibition or disruption reduced Hh signaling in GCPs [2], it will be essential to determine whether the APC/C-CK1δ interaction is important for Hh signaling in GCPs. Future studies will determine the importance of the APC/C-CK1δ interaction in various signaling pathways including Hh and WNT, where CK1δ has been implicated [1]. Furthermore, it will be critical to determine whether the APC/C-CK1δ interaction is dysregulated in various neurological diseases.
